# The Use of Antihypertensive Medication and the Risk of Breast Cancer in a Case-Control Study in a Spanish Population: The MCC-Spain Study

**DOI:** 10.1371/journal.pone.0159672

**Published:** 2016-08-10

**Authors:** Inés Gómez-Acebo, Trinidad Dierssen-Sotos, Camilo Palazuelos, Beatriz Pérez-Gómez, Virginia Lope, Ignasi Tusquets, M. Henar Alonso, Victor Moreno, Pilar Amiano, Antonio José Molina de la Torre, Aurelio Barricarte, Adonina Tardon, Antonio Camacho, Rosana Peiro-Perez, Rafael Marcos-Gragera, Montse Muñoz, Maria Jesus Michelena-Echeveste, Luis Ortega Valin, Marcela Guevara, Gemma Castaño-Vinyals, Nuria Aragonés, Manolis Kogevinas, Marina Pollán, Javier Llorca

**Affiliations:** 1 CIBER Epidemiologia y Salud Pública (CIBERESP), Madrid, Spain; 2 University of Cantabria–IDIVAL, Santander, Spain; 3 Cancer and Environmental Epidemiology Unit, National Center for Epidemiology, Carlos III Institute of Health, Madrid, Spain; 4 Cancer Epidemiology Research Group, Oncology and Hematology Area, IIS Puerta de Hierro (IDIPHIM), Madrid, Spain; 5 Servei d'Oncologia Mèdica, Hospital del Mar, Barcelona, Spain; 6 Cancer Research Program IMIM (Hospital del Mar Medical Research Institute), Barcelona, Spain; 7 Universitat Autònoma de Barcelona, Barcelona, Spain; 8 Cancer Prevention and Control Program, Catalan Institute of Oncology-IDIBELL, and University of Barcelona, Barcelona, Spain; 9 Public Health Division of Gipuzkoa, Biodonostia Research Institute, Guipuzkoa, Spain; 10 Área de Medicina Preventiva y Salud Pública, Departamento de Ciencias Biomédicas, Universidad de León, León, España; 11 Grupo de Investigación en Interacciones Gen-Ambiente y Salud (GIIGAS), Universidad de León, León, España; 12 Navarra Public Health Institute, Pamplona, Spain; 13 Navarra Institute for Health Research (IdiSNA) Pamplona, Pamplona, Spain; 14 IUOPA, Universidad de Oviedo, Oviedo, Spain; 15 Hospital Juan Ramon Jimenez, Andalusian Health Service, Huelva, España; 16 Research Center for Health and the Environment (CYSMA), Universidad de Huelva, Huelva, España; 17 Area de Cáncer y Salud Pública, Fundación FISABIO- Salud Pública, Valencia, Spain; 18 Epidemiology Unit and Girona Cancer Registry, Oncology Coordination Plan, Department of Health, Autonomous Government of Catalonia and Descriptive Epidemiology, Genetics and Cancer Prevention Group [Girona Biomedical Research Institute (IdIBGi)], Catalan Institute of Oncology, Girona, Spain; 19 Translational Genomics and Targeted Therapeutics in Solid Tumors (IDIBAPS), Barcelona, Spain; 20 Onkologikoa- Oncology Institute Gipuzkoa, Guipuzkoa, Spain; 21 Pharmacy Service, Complex assistive university Leon, Leon, Spain; 22 Navarra Institute for Health Research (IdiSNA), Pamplona, Spain; 23 Centre for Research in Environmental Epidemiology (CREAL), Barcelona, Spain; 24 Universitat Pompeu Fabra (UPF), Barcelona, Spain; 25 IMIM (Hospital del Mar Medical Research Institute), Barcelona, Spain; University of North Carolina at Chapel Hill School of Medicine, UNITED STATES

## Abstract

**Introduction:**

The evidence on the relationship between breast cancer and different types of antihypertensive drugs taken for at least 5 years is limited and inconsistent. Furthermore, the debate has recently been fueled again with new data reporting an increased risk of breast cancer among women with a long history of use of antihypertensive drugs compared with nonusers.

**Methods:**

In this case-control study, we report the antihypertensive drugs–breast cancer relationship in 1,736 breast cancer cases and 1,895 healthy controls; results are reported stratifying by the women’s characteristics (i.e., menopausal status or body mass index category) tumor characteristics and length of use of antihypertensive drugs.

**Results:**

The relationship among breast cancer and use of calcium channel blockers (CCB) for 5 or more years had odds ratio (OR) = 1.77 (95% CI, 0.99 to 3.17). Stratifying by BMI, the OR increased significantly in the group with BMI ≥ 25 (OR 2.54, 95% CI, 1.24 to 5.22). CCBs were even more strongly associated with more aggressive tumors, (OR for invasive tumors = 1.96, 95% CI = 1.09 to 3.53; OR for non ductal cancers = 3.97, 95% CI = 1.73 to 9.05; OR for Erbb2+ cancer = 2.97, 95% CI: 1.20 to 7.32). On the other hand, premenopausal women were the only group in which angiotensin II receptor blockers may be associated with breast cancer (OR = 4.27, 95% CI = 1.32 to 13.84) but this could not be identified with any type or stage. Use of angiotensin-converting-enzyme inhibitors, beta blockers and diuretics were not associated with risk.

**Conclusions:**

In this large population-based study we found that long term use of calcium channel blockers is associated with some subtypes of breast cancer (and with breast cancer in overweight women).

## Introduction

Hypertension is a highly prevalent disease affecting around 30–45% of the general population [1] and antihypertensive medications are among the most commonly prescribed medications. According to the latest data provided by the International Marketing Services (IMS), consumption of antihypertensive drugs in Spain has tripled in the last 15 years [2]. Moreover, once established, antihypertensive drugs are usually given for the rest of the patient’s life and the number of antihypertensive drugs available is increasing.

Breast cancer is the most common cancer among women in both developed and developing countries. One in ten of all new cancers diagnosed worldwide each year is a cancer of the female breast. It is also the leading cause of cancer death among women worldwide. More than 1.67 million cases are diagnosed and more than 522,000 patients die from it worldwide every year [3].

The carcinogenic potential of antihypertensive drugs has been debated for nearly 50 years [4]. Even since the nineties, contradictions between different studies have been observed. Some studies showed that calcium channel blockers (CCBs) increase the overall risk of cancer, but no significant association was found with breast cancer [5,6]. Other studies observed that CCBs specifically increase the risk of breast cancer [7–9]; in contrast to others that did not find such association [10–15]. The debate has recently been fueled again with new data reporting an increased risk of breast cancer among women with a long history of use of antihypertensive drugs compared with nonusers [16–19].

The discrepant results and the high prevalence of antihypertensive medication in middle-aged population justify carrying out new research in order to provide additional evidence about the relationship with cancer development. The aim of the present study is to assess the association between breast cancer and previous use of antihypertensive medication, taking into account the class of antihypertensive drug and the duration of use, in a large population-based case-control study conducted in Spain, the MCC-Spain study.

## Materials and Methods

### Ethics Statement

This study was approved by the corresponding ethics committee of each area (Comité ético de investigación clínica de Asturias, Barcelona, Cantabria, Girona, Gipuzkoa, Huelva, León, Madrid, Navarra and Valencia) and informed written consent was obtained from parents. The MCC-Spain study also followed the Declaration of Helsinki and the Spanish Personal Data Protection Act of 1999.

### Study design and population

The Multi Case-Control (MCC-Spain) study has been described in detail [20]. Briefly, it is a population-based case-control study of common tumors in Spain; the recruitment includes incident cases of colorectal, breast, gastroesophageal and prostate cancer diagnosed between September 1st, 2008 and December 31st, 2013. Henceforth, we will only refer to breast cancer cases and their controls.

All cases of breast cancer included were incident and pathology confirmed, with no previous diagnosis of breast cancer; they were aged between 20 and 85 years old, and resident within the influence area of the hospital for at least 6 months prior to recruitment in 10 Spanish provinces (Asturias, Barcelona, Cantabria, Girona, Gipuzkoa, Huelva, León, Madrid, Navarra and Valencia). Controls with no prior history of breast cancer were selected from the general population according to age and regional distribution of the cases included in the study. In this paper, 1736 cases of breast cancer in women and their 1895 frequency-matched controls were considered. Response rates were 71% for breast cancer and 72% for controls, with no differences in the main socio-demographic variables among those who participated and those who refused to participate.

### Exposure data

Participants were interviewed face-to-face by trained interviewers, using a comprehensive epidemiological questionnaire that collected socio-demographic information, personal and family history of cancer, anthropometric data, smoking habits, alcohol intake, occupation, physical activity, water consumption, reproductive and medical history and medication use, family history, sun exposure, sleep habits, use of hygiene products and cosmetics, signs and symptoms. Comprehensive dietary habits were obtained with the use of a validated food-frequency questionnaire.

Participant’s weight was self-reported, as estimated one year before diagnosis for cases and one year before the interview for controls. Accordingly, body mass index (BMI) was calculated considering self-reported weight, referred to that date, and height. Total fat and vegetable intakes were estimated from the questionnaire using local food composition tables. Similar estimates provided total energy consumption. Physical activity was recorded for the longest occupation and also considering recreational physical exercise.

Detailed information was obtained on past medical conditions and the corresponding medications used. The age at onset, the dates of diagnosis or occurrence and the type of treatment received for each condition was also registered.

### Drug use assessment

Drug use was recorded by indication. For each drug, the brand name, dose and duration of exposure were recorded to identify patients with regular drug consumption (“no” and “occasionally” versus “yes”) and the duration of consumption.

The drugs were coded following the Anatomical Therapeutic Chemical Classification System (ATC codes) to define groups with similar mechanisms of action [WHOCC Homepage. WHO Collaborating Centre for Drug Statistics Methodology].

All drugs indicated for the treatment of hypertensive diseases have been considered. The ATC codes included in the present analysis are code C02 (Antihypertensive), C03 (Diuretics), C04 (Peripheral vasodilators), C07 (Beta blocking agents), C08 (Calcium channel blockers) and C09 (Agents acting on the renin-angiotensin system). Results will be presented for each separate group and for specific antihypertensive drugs that had a prevalence of use over 1% in controls.

### Statistical Methods

Unconditional binomial logistic regression was used to assess the association between antihypertensive drug use and breast cancer overall and stratifying for menopausal status and BMI (<25/≥25 kg/m^2^). In order to study the relationship between antihypertensive drug use and different breast cancer subtypes, we applied multinomial logistic regression models; multinomial logistic regression is useful when the outcome is categorical rather than dichotomic; for instance, according to cancer stage, participants were classified in one out of three categories: control / breast cancer stage I-II / breast cancer stage III-IV. Multinomial logistic regression allows to estimate odds ratios for every category (i.e.: breast cancer stage I-II / breast cancer stage III-IV) comparing with the reference category (i.e.: control) [21]. Statistical models were adjusted for the following confounders: age, area of residence, education, BMI 1 year before, active smoking, alcohol intake in the past, family history of breast cancer, age of menarche, age at first full-term birth, parity, menopausal status and hormonal therapy.

Stratified models were developed according to menopausal status, BMI (<25/≥25 kg/m^2^), clinical stage (I-II / III-IV), ductal (ductal/non ductal), invasive and immunohistochemistry (hormone + receptors with Erbb2 negative, Erbb2 + receptors and triple negative receptors). Results, which are reported only in strata with at least 5 cases or controls using antihypertensive drugs are shown as odds ratios (OR) with 95% confidence intervals (CI). All reported p-values are two-tailed. Statistical analysis was carried out using the package Stata 14/SE (StataCorp, College Station, Tx, US).

## Results

There were 1736 cases of breast cancer and 1895 controls. Table 1 describes the characteristics of the women participating in this study. Compared with women in the control group, cases were younger (56.4 Vs 59.0), used to smoke more (former smoker 26% cases, 21% controls) though the proportion of current smokers was similar in both groups, had undergone fewer deliveries (1.9 vs 2.0) and were more likely to have family history of breast cancer. The proportion of premenopausal women was higher in cases than in controls (40% vs 33%). With respect to food, cases consumed more kilocalories per day (1861 versus 1754). The grams per day of red meat and alcohol were also higher in the group of women with breast cancer.

**Table 1:** Main characteristics of cases and controls from the study population (only women have been included).

Baseline and clinical characteristics		Category	Breast Cancer Cases	Population Controls	p
			N = 1736	N = 1895	
Age, mean±sd			56.4±12.6	59.0±13.2	<0.001
Geographical area, n (%)		Asturias	70 (4.0)	121 (6.4)	<0.001
		Barcelona	292 (16.8)	380 (20.1)	
		Cantabria	141 (8.1)	188 (9.9)	
		Girona	47 (2.7)	57 (3.0)	
		Gipuzkoa	226 (13.0)	255 (13.5)	
		Huelva	105 (6.1)	79 (4.2)	
		Leon	227 (13.1)	202 (10.7)	
		Madrid	341 (19.6)	365 (19.3)	
		Navarra	226 (13.0)	181 (9.6)	
		Valencia	61 (3.5)	67 (3.5)	
Antihyperstensive drug consumption, n (%)	Any antihypertensive therapy	Yes	364(21.0)	406 (21.4)	0.651
		No	1372(79.0)	1489(78.6)	
	Diuretics	Yes	101(5.8)	111(5.8)	0.996
		No	1635(94.2)	1798(94.2)	
	Calcium channel blockers	Yes	61(3.5)	58(3.0)	0.42
		No	1675(96.5)	1851(97.0	
	B-blockers	Yes	76(4.4)	86(4.5)	0.852
		No	1660(95.6)	1823(95.5)	
	Angitensin-converting-enzyme inhibitors [ACEIs]	Yes	131(7.6)	160(8.4)	0.353
		No	1605(92.5)	1749(91.6)	
	Angiotensin II receptor blockers [ARBs]	Yes	129(7.4)	133(7.0)	0.588
		No	1607(92.6)	1776(93.0	
Family history of breast cancer, n (%)		No	1288(74.2)	1614 (85.2)	<0.001
		First-degree relative	256 (14.8)	166 (8.8)	
		Second-degree relative	174 (10.0)	105 (5.5)	
		Not Available	18 (0.8)	10(0.5)	
Educational level, n (%)		Less than primary school	268 (15.4)	327 (17.3)	0.1
		Primary school	565 (32.6)	581 (30.7)	
		Secondary school	573 (33.0)	585 (30.9)	
		University	330 (19.0)	402 (21.2)	
Tobacco smoking, n (%)		Never smoker	972 (56.0)	1141 (60.2)	0.002
		Former smoker	450 (25.9)	397 (21.0)	
		Current smoker	314 (18.1)	357 (18.8)	
Body Mass Index (kg/m^2^), n (%)		<18.5	30 (1.7)	43 (2.3)	0.31
		18.5–24.9	789 (45.5)	899 (47.4)	
		25.0–29.9	590 (34.0)	601 (31.7)	
		≥30	327 (18.8)	352 (18.6)	
Energy intake (kcal/day), mean±sd			1861±644	1754±566	<0.001
Ethanol intake in the past (g/day), mean±sd			6.2±11.5	5.3±9.5	0.01
Red meat intake (g/day), mean±sd			26.9±20.2	25.2±19.9	0.01
Fruit intake (g/day), mean±sd			363±239	365±222	0.87
Vegetable intake (g/day), mean±sd			196±133	198±119	0.6
Number of full-term, mean±sd			1.9±1.5	2.0±1.6	0.03
Menopausal status, n (%)		Premenopausal	702 (40.4)	628 (33.1)	<0.001
		Postmenopausal	1034 (59.6)	1267 (66.9)	
Age at first full-term, mean±sd*			26.5±5.0	26.5±4.7	0.82
Age at menarche, mean±sd			12.8±1.5	12.9±1.5	0.02
Age at menopause, mean±sd			48.8±5.4	48.5±5.3	0.18
Previous use of hormonal contraceptives, n (%)			789 (45.5)	868 (45.8)	0.83

*exclude nulliparous

Clinical-pathological characteristics of the breast cancers are reported in Table 2; ductal cancer accounts for 74% cases; two out of three breast cancers were diagnosed at stage I or II; more than 60% cancers were hormonal receptors, 14% were Erbb2 receptors + and only 9% were triple negative breast cancers.

**Table 2:** Clinical and pathological characteristics of breast cancers.

Classification	N (%)
Pathology	
Ductal	1289 (74.3)
Lobular	112 (6.5)
Papilar	22 (1.3)
Coloid	20 (1.2)
Tubular	12 (0.7)
Mixed	27 (1.6)
Other	35 (2.0)
Not Available	213 (12.3)
Clinical stage	
0	115 (6.6)
I	604 (34.8)
II	495 (28.5)
III	182 (10.5)
IV	22 (1.3)
Not Available	318 (18.3)
Invasive	
Invasive	1497 (86.2)
Non-invasive	166 (9.6)
Not Available	73 (4.2)
Inmunohistochemistry	
Hormonal receptors	1117 (64.3)
Erbb2+	255 (14.7)
Triple –	157 (9.04)
Not Available	207(11.9)

### Antihypertensive drug consumption and risk of breast cancer according to women’s characteristics

Table 3 and S1 Table–displaying the duration of consumption- show the relationship between the use of antihypertensive drugs and the risk of breast cancer overall- and stratified by menopausal status and BMI. No significant associations were found between breast cancer and any antihypertensive drug for all women combined (Table 3). Users of any antihypertensive drug doubled the risk of developing breast cancer in premenopausal women (OR = 2.15, 95% CI = 1.17 to 3.96).

**Table 3:** Relationship between antihypertensive drug consumption and breast cancer according to women’s characteristics. Category reference no antihypertensive treatment.

			Population Controls	Breast Cancer Cases if antihypertensive therapy consumption				
			Exp / UnExp	Exp / UnExp	*Adjusted* ^a^OR	*95% CI*		*p-value*
*any antihypertensive therapy*	*All women*		367/1497	323/1372	1.16	0.94	1.43	0.17
* *	Menopausal *	*Premenopausal*	**27/596**	**46/654**	**2.15**	**1.17**	**3.96**	**0.014**
* *	* *	*postmenopausal*	335/893	277/718	1.09	0.86	1.37	0.468
* *	*BMI*	*<25*	120/809	74/742	1.04	0.71	1.54	0.838
* *	* *	*≥25*	247/688	249/630	1.19	0.92	1.54	0.179
*Diuretics*	*All women*	* *	104/1798	91/1635	0.98	0.7	1.39	0.929
* *	Menopausal *	*Premenopausal*	8/619	9/692	1.26	0.41	3.93	0.687
* *	* *	*postmenopausal*	95/1165	82/943	1	0.69	1.45	0.991
* *	*BMI*	*<25*	30/911	15/803	0.66	0.3	1.43	0.289
* *	* *	*≥25*	74/887	76/832	1.08	0.73	1.59	0.713
*Calcium Channel Blockers*	*All women*	* *	52/1851	53/1675	1.56	0.98	2.48	0.063
* *	Menopausal *	*Premenopausal*	3/624	3/699	0.51	0.05	5.15	0.567
* *	* *	*postmenopausal*	**48/1213**	**50/976**	**1.72**	**1.05**	**2.8**	**0.03**
* *	*BMI*	*<25*	19/921	10/808	0.89	0.34	2.3	0.81
* *	* *	*≥25*	**33/930**	**43/867**	**2.05**	**1.16**	**3.63**	**0.013**
*B- blockers*	*All women*	* *	78/1823	63/1660	1.11	0.75	1.63	0.614
* *	Menopausal *	*Premenopausal*	9/619	7/695	1.1	0.35	3.42	0.869
* *	* *	*postmenopausal*	67/1191	56/965	1.15	0.75	1.75	0.515
* *	*BMI*	*<25*	27/911	16/803	1.3	0.64	2.65	0.468
* *	* *	*≥25*	51/912	47/857	1	0.62	1.61	0.99
*Angiotensin-converting-enzyme inhibitors [ACEIs]*	*All women*	* *	136/1749	116/1605	1.02	0.75	1.38	0.918
* *	Menopausal *	*Premenopausal*	8/615	14/687	1.61	0.6	4.34	0.346
* *	* *	*postmenopausal*	125/1122	102/918	1	0.72	1.38	0.977
* *	*BMI*	*<25*	51/887	23/795	0.65	0.35	1.2	0.167
* *	* *	*≥25*	85/862	93/810	1.17	0.81	1.69	0.406
*Angiotensin II receptor blockers [ARBs]*	*All women*	* *	124/1776	118/1607	1.19	0.87	1.62	0.286
* *	Menopausal *	*Premenopausal*	**8/620**	**18/683**	**4.27**	**1.32**	**13.84**	**0.015**
* *	* *	*postmenopausal*	115/1144	100/924	1.05	0.75	1.46	0.788

Abbreviations: CI, Confidence interval; OR, odds ratio

^a^OR adjusted for the matching factors age, area of resident, education, body mass index, active smoking, alcohol intake, family history of breast cancer, age of menarche, age first full-term births, number of full-term births, menopausal status, hormonal therapy.

* OR adjusted for the matching factors age, area of resident, education, body mass index, active smoking, alcohol intake, family history of breast cancer, age of menarche, age first full-term births, number of full-term births, hormonal therapy

When examining specific classes of antihypertensive drugs, the use of CCBs was associated with a 72% increased risk of breast cancer in the postmenopausal group (OR = 1.72, 95% CI = 1.05 to 2.80) and twice the risk in women with BMI ≥ 25 (OR = 2.05, 95% CI = 1.16 to 3.63); there is moderate confirmation of both associations in women taking CCBs for 5 years or more. Angiotensin II receptor blockers (ARB) were the only group associated with an increased risk of breast cancer in premenopausal women (OR = 4.27, 95% CI = 1.32 to 13.84), but not in postmenopausal women (p for ARB–menopausal status interaction = 0.03). The use of diuretics, beta blockers and angiotensin converting enzyme inhibitors (ACEI) was not associated with breast cancer risk in any women or in different strata.

### Antihypertensive drug consumption and risk of breast cancer according to tumor characteristics

Results of the association between the use of antihypertensive drugs and incident breast cancer according to tumor characteristics are shown in Table 4 and in S2 Table for the duration of consumption. Altogether, the use of antihypertensive drugs was associated with a higher risk of triple negative breast cancer (OR = 2.21, 95% CI = 1.37 to 3.56; p for heterogeneity = 0.03); this result was consistently reproduced in women undergoing antihypertensive treatment for more or less than 5 years (S2 Table). Antihypertensive drugs were also associated with more aggressive or worse prognosis cancer: taking antihypertensive drugs increased the risk of developing a tumor in clinical stage III-IV (OR = 1.62; 95% CI = 1.04 to 2.52; p for antihypertensive drug–clinical stage heterogeneity = 0.22), non-ductal tumor (OR = 1.49; 95% CI = 1.00 to 2.24; p for antihypertensive drug–ductal cancer interaction = 0.32), invasive cancer (OR = 1.26, 95% CI = 1.01 to 1.57; p for antihypertensive drug–invasive cancer interaction = 0.02), although results taking into account the treatment length did not reveal a risk pattern consistent with higher risk in women taking antihypertensive drugs for more than 5 years (S2 Table).

**Table 4:** Relationship between antihypertensive drug consumption and breast cancer according to tumor characteristic. Category reference: no antihypertensive treatment.

			*Population*	*Breast Cancer Cases if antihypertensive therapy consumption*				
			*Controls*	* *	* *	* *	* *	* *
			Exp / UnExp	Exp / UnExp	*Adjusted* ^a^OR	*95% CI*	* *	*p-value*
*any antihypertensive therapy*	*Clinical Stage*	I-II	367/1497	208/872	1.22	0.96	1.55	0.105
* *	* *	III-IV	**367/1497**	**47/154**	**1.62**	**1.04**	**2.52**	**0.032**
* *	*ductal*	Ductal	367/1497	245/1015	1.21	0.96	1.53	0.103
* *	* *	Non ductal	**367/1497**	**50/177**	**1.49**	**1**	**2.24**	**0.052**
* *	*Invasive*	In situ	367/1497	21/143	0.63	0.35	1.13	0.123
* *	* *	Invasive	**367/1497**	**289/1172**	**1.26**	**1.01**	**1.57**	**0.038**
* *	*Inmunohistochemistry*	hormone +receptors	367/1497	210/884	1.13	0.89	1.44	0.313
* *	* *	Erbb2+	367/1497	45/205	1.13	0.74	1.73	0.563
* *	* *	receptors						
* *	* *	triple negative receptors	**367/1497**	**41/111**	**2.21**	**1.37**	**3.56**	**0.001**
*Diuretics*	*Clinical Stage*	I-II	104/1798	55/1039	0.9	0.6	1.34	0.598
* *	* *	III-IV	104/1798	11/193	1	0.48	2.09	0.994
* *	*ductal*	Ductal	104/1798	72/1209	1.03	0.71	1.49	0.89
* *	* *	Non ductal	104/1798	10/223	0.78	0.37	1.63	0.507
* *	*Invasive*	In situ	104/1798	5/161	0.68	0.24	1.95	0.473
* *	* *	Invasive	104/1798	82/1406	1.01	0.71	1.45	0.939
* *	*Inmunohistochemistry*	hormone +receptors	104/1798	56/1054	0.9	0.6	1.34	0.601
* *	* *	Erbb2+	104/1798	14/240	1.05	0.54	2.07	0.881
* *	* *	receptors						
* *	* *	triple negative receptors	104/1798	13/144	1.54	0.77	3.08	0.224
*Calcium Channel Blockers*	*Clinical Stage*	I-II	52/1851	29/1065	1.34	0.78	2.3	0.287
* *	* *	III-IV	**52/1851**	**11/193**	**2.7**	**1.23**	**5.95**	**0.014**
* *	*ductal*	Ductal	52/1851	38/1248	1.5	0.9	2.51	0.12
* *	* *	Non ductal	**52/1851**	**12/218**	**2.63**	**1.27**	**5.43**	**0.009**
* *	*Invasive*	In situ	52/1851	3/163	-	-	-	-
* *	* *	Invasive	**52/1851**	**49/1441**	**1.67**	**1.04**	**2.7**	**0.035**
* *	*Inmunohistochemistry*	hormone +receptors	52/1851	33/1080	1.46	0.86	2.47	0.164
* *	* *	Erbb2+	**52/1851**	**12/242**	**2.52**	**1.18**	**5.37**	**0.017**
* *	* *	receptors						
* *	* *	triple negative receptors	52/1851	4/152	-	-	-	.
*B- blockers*	*Clinical Stage*	I-II	78/1823	40/1054	1.1	0.71	1.72	0.658
* *	* *	III-IV	78/1823	9/194	1.23	0.54	2.8	0.626
* *	*ductal*	Ductal	78/1823	48/1229	1.16	0.76	1.77	0.5
* *	* *	Non ductal	78/1823	8/225	1.04	0.48	2.26	0.921
* *	*Invasive*	In situ	78/1823	4/162	-	-	-	.
* *	* *	Invasive	78/1823	55/1429	1.17	0.78	1.75	0.455
* *	*Inmunohistochemistry*	hormone +receptors	78/1823	38/1073	0.96	0.61	1.52	0.876
* *	* *	Erbb2+receptors	78/1823	6/246	0.79	0.33	1.9	0.602
* *	* *	triple negative receptors	78/1823	9/146	2.04	0.95	4.38	0.068
*Angiotensin-converting-enzyme inhibitors [ACEIs]*	*Clinical Stage*	I-II	136/1749	77/1015	1.09	0.77	1.53	0.634
* *	* *	III-IV	136/1749	14/189	1.01	0.51	1.98	0.982
* *	*ductal*	Ductal	136/1749	83/1196	0.95	0.67	1.33	0.754
* *	* *	Non ductal	**136/1749**	**22/209**	**1.78**	**1.06**	**2.98**	**0.029**
* *	*Invasive*	In situ	136/1749	9/157	0.86	0.38	1.95	0.725
* *	* *	Invasive	136/1749	104/1380	1.1	0.8	1.51	0.554
* *	*Inmunohistochemistry*	hormone +receptors	136/1749	85/1025	1.19	0.85	1.67	0.321
* *	* *	Erbb2+receptors	136/1749	12/242	0.7	0.35	1.39	0.303
* *	* *	triple negative receptors	136/1749	14/139	1.43	0.73	2.79	0.293
*Angiotensin II receptor blockers [ARBs]*	*Clinical Stage*	I-II	124/1776	76/1019	1.34	0.95	1.9	0.099
* *	* *	III-IV	124/1776	19/184	1.75	0.97	3.18	0.065
* *	*ductal*	Ductal	124/1776	91/1189	1.28	0.91	1.79	0.152
* *	* *	Non ductal	124/1776	16/218	1.22	0.67	2.21	0.514
* *	*Invasive*	In situ	124/1776	9/155	0.71	0.29	1.73	0.452
* *	* *	Invasive	124/1776	104/1384	1.24	0.9	1.71	0.197
* *	*Inmunohistochemistry*	hormone +receptors	124/1776	74/1039	1.11	0.78	1.58	0.568
* *	* *	Erbb2+	124/1776	19/234	1.45	0.81	2.61	0.214
* *	* *	receptors						
* *	* *	triple negative receptors	124/1776	14/142	1.74	0.88	3.41	0.109

Abbreviations: CI, Confidence interval; OR, odds ratio

^a^OR adjusted for the matching factors age, area of resident, education, body mass index, active smoking, alcohol intake, family history of breast cancer, age of menarche, age first full-term births, number of full-term births, menopausal status, hormonal therapy.

Looking at specific classes of antihypertensive drugs, CCBs were even more strongly associated with more aggressive tumors, multiplying by 2.7 the risk of tumors in stage III-IV (OR = 2.70, 95% CI = 1.23 to 5.95), non-ductal cancers (OR = 2.63, 95% CI = 1.27 to 5.43), and Erbb2+ cancer (OR = 2.52, 95% CI: 1.18 to 5.37). CCBs were also associated with invasive tumors (OR = 1.67, 95% CI = 1.04 to 2.70). Similar results were found in women taking CCBs for at least five years.

The use of diuretics, beta blockers, ACEIs or ARBs was not associated with increased risk for specific tumor characteristics.

## Discussion

In this population-based case-control study the use of antihypertensive medications as a global group was associated with higher risk of invasive breast cancer, and this risk appears to be confined to triple negative breast cancer and concentrated in premenopausal women. Our results were similar to those found in a large prospective study, the California Teachers Study (CTS) with 133,479 women [16]. In contrast, another recent study, the Nurses’ Health Study (NHS) with 210,641 participants [22], did not find this association. On the other hand, we found that CCB consumption increased the odds of breast cancer in postmenopausal women, women with BMI over 25 kg/m^2^, cancer in stages III-IV, non-ductal cancer and Erbb2+ cancer. Previous results on CCB-breast cancer relationship have been contradictory; Fitzpatrick et al, in a study limited to women aged 65 years or more, found an elevated risk of breast cancer associated with CCB usage [7]; Li et al (2003) reported an increase in breast cancer risk in former users of CCBs, but they failed to find any trend of increasing risk associated with longer duration [8]; Li et al (2013) found that CCB usage was associated with both ductal and non-ductal breast cancer, but only if duration of CCB consumption was longer than 10 years [17]; while a small but statistically significant effect of CCBs on breast cancer incidence was also reported by Leung (2015)[18]. Negative results have been published, however, in other studies [10–15,23,24].

CCBs may increase the risk of cancer by changing intracellular calcium levels, which could affect the process of programmed cell death, not enabling the destruction of damaged cells to prevent the development of diseases such as cancer, resulting in indiscriminate replication of an impaired cell [25]. Calcium plays a regulatory role in apoptosis acting through various signaling pathways such as the activation of the caspase [26–28] or the induction of endonuclease activity [29]. In addition, calcium is involved in triggering cell death by mitochondrial permeabilization [30] and promoting phagocytosis by phosphatidylserine exposure on the cell surface by apoptosis [31].

On the other hand, nifedipine–a CCB- has been found to increase proliferation and migration of breast cancer cells, which could be responsible for the association between CCBs and late stage cancers. This nifedipine effect–which is not shared by other CCBs such as verapamil- seems to be produced via the Erk pathway activation and is independent of the calcium channel-blocking effect [32].

### Other antihypertensive drugs

ARBs were the only antihypertensive group associated with an increased risk of breast cancer in premenopausal women in our results; using ARBs before menopause was, however, scarce; therefore, when stratifying by length of consumption the results were non-significant, although the odds ratios scaled from 3.48 for less than 5 year users to 6.64 for 5 or more year users. We have not found other papers analyzing the relationship between ARBs and breast cancer in premenopausal women; this together with the small number of premenopausal women taking ARBs make the interpretation of this result highly speculative. Bhashkaran et al (2012) found an increased risk of breast cancer associated with short-term exposure to ARBs; they suggested that such an association could not be causal but the result of confounding by indication: according to them, some early symptoms of breast cancer could induce ARBs to be indicated instead of ACEIs in hypertensive patients, leading, therefore, to a spurious ARB–breast cancer association [33]. We have no data for exploring such an explanation in our study.

Differences in study design and population characteristics may explain the conflicting results reported on the antihypertensive drugs–breast cancer association. Inferring is difficult because of the small sample size in some studies, differences in the populations evaluated and designs (cohort of patients, general population cohorts and case-control studies). In many cases, randomized trials cannot identify long-term adverse effects of medication because they are usually conducted for relatively shorter periods (i.e.: 5 years or less) [34]. Subsequent long-term monitoring of drugs through observational studies may overcome this limitation and provide new information in this regard. Some observational studies have analyzed the antihypertensive drugs-breast cancer relationship by working with administrative data, which were not designed for this objective. That kind of design does not allow an adequate adjustment for confounding factors. Although some studies linked the use of CCB and cancer in the 90s, antihypertensive consumption was different to today’s. Nowadays, more therapeutic options are available; the use of antihypertensive drugs is characterized by the appearance of new fixed-dose combinations of two active antihypertensive drugs and by the introduction of new drug treatments belonging to the group of ARBs, beta blockers or CCBs [1].

The present study has some limitations. First, recall bias should be considered as in any case-control study. Drug consumption was obtained using a standardized questionnaire in face-to-face interviews where examiners were blinded to the case-control status. If a non-differential recall bias was produced, then the odds ratios should be downward biased and the positive associations we have found for CCB, ARBs or ACEIs would actually be even stronger than reported here. If a differential recall bias were responsible for these associations, breast cancer cases would also have over-declared (or controls under-declared) their consumption of other hypertensive drugs; however, no association has been found between breast cancer and diuretics or beta-blockers, which makes a differential recall bias less probable. Second, some strata in our study have small numbers of exposed cases or controls; this could produce unstable estimates. This limitation should be especially considered regarding ARBs, as their relationship with breast cancer is confined to premenopausal women, a relatively small subgroup whose exposure to hypertensive drugs could not be too long. Third, antihypertensive consumption was reported by indication; therefore, most people taking antihypertensive drugs have hypertension. Thus, we cannot distinguish using antihypertensive drugs from having hypertension. Lastly, many comparisons have been made, raising the probability of finding some spurious results However, the consistency of some results in different subgroups, such as those for CCBs, supports the existence of a real excess risk associated with their use.

In summary, we report that consumption of antihypertensive medications -as a global group- was associated with an increased odds of breast cancer in premenopausal women, and the use of CCBs in particular was associated with an increased odds of breast cancer in postmenopausal women and those with BMI higher than 25 Kg/m^2^. As people with hypertension are expected to take antihypertensive drugs for many years, their relative effect on breast cancer should be taken into account when choosing the antihypertensive to be prescribed.

## Supporting Information

S1 Table Association between duration of antihypertensive drug consumption (<5 years and ≥5 years) and the risk of breast cancer according to women’s characteristic. Category reference no antihypertensive treatment.
(DOCX)

S2 Table Association between duration of antihypertensive drug consumption (<5 years and ≥5 years) and the risk of breast cancer according to characteristics of tumor and immunohistochemistry. Category reference no antihypertensive treatment.
(DOCX)

## Abbreviations

OR: Odds ratios
CI: Confidence intervals
CCBs: Calcium channel blockers
ARBs: Angiotensin II receptor blockers
ACEIs: Angiotensin-converting-enzyme inhibitors
MCC: Spain: Multi Case-Control Spain
