# Supplementary material for: The Use of Antihypertensive Medication and the Risk of Breast Cancer in a Case-Control Study in a Spanish Population: The MCC-Spain Study
Source: PLoS One. 2016 Aug 10;11(8):e0159672. doi: 10.1371/journal.pone.0159672 (PMC4979888; doi:10.1371/journal.pone.0159672)
Supplement: S1 Table — Category reference no antihypertensive treatment. (DOCX) [file pone.0159672.s001.docx]

**S1 Table. Association between duration of antihypertensive drug consumption (<5 years and ≥5 years) and the risk of breast cancer according to women’s characteristic. Category reference no antihypertensive treatment**

|  |  |  | **Population Controls** | **Breast Cancer Cases if antihypertensive therapy use <5 years** | | | | | **Population Controls** | **Breast Cancer Cases if antihypertensive therapy use ≥5 years** | | | | |
| --- | --- | --- | --- | --- | --- | --- | --- | --- | --- | --- | --- | --- | --- | --- |
|  |  |  | **Exp /UnExp** | **Exp /UnExp** | ***Adjusted OR*** | ***95% CI*** | | ***p-value*** | **Exp /UnExp** | **Exp /UnExp** | ***Adjusted OR*** | ***95% CI*** | | ***p-value*** |
| ***any antihypertensive therapy*** | ***All women*** |  | 142/1497 | 132/1372 | 1.25 | 0.92 | 1.68 | 0.153 | 225/1497 | 191/1372 | 1.11 | 0.86 | 1.43 | 0.44 |
|  | ***Menopausal **** | ***Premenopausal*** | **15/596** | **26/654** | **2.28** | **1.06** | **4.93** | **0.036** | 12/596 | 20/654 | 1.96 | 0.77 | 4.96 | 0.155 |
|  |  | ***postmenopausal*** | 125/893 | 106/718 | 1.13 | 0.81 | 1.58 | 0.466 | 210/896 | 171/718 | 1.06 | 0.81 | 1.4 | 0.651 |
|  | ***BMI*** | ***<25*** | 53/809 | 36/742 | 1.36 | 0.79 | 2.35 | 0.264 | 67/809 | 38/742 | 0.84 | 0.51 | 1.39 | 0.494 |
|  |  | ***≥25*** | 89/688 | 96/630 | 1.19 | 0.82 | 1.71 | 0.357 | 158/688 | 153/630 | 1.2 | 0.88 | 1.62 | 0.248 |
| ***Diuretics*** | ***All women*** |  | 45/1798 | 42/1635 | 1.07 | 0.66 | 1.75 | 0.777 | 59/1798 | 49/1635 | 0.91 | 0.58 | 1.45 | 0.699 |
|  | ***Menopausal **** | ***Premenopausal*** | 6/619 | 7/692 | 1.64 | 0.43 | 6.2 | 0.469 | 2/619 | 2/692 | 1 | 1 | 1 | . |
|  |  | ***postmenopausal*** | 39/1165 | 35/943 | 1.08 | 0.63 | 1.85 | 0.781 | 56/1165 | 47/943 | 0.95 | 0.59 | 1.53 | 0.821 |
|  | ***BMI*** | ***<25*** | 16/911 | 6/803 | 0.51 | 0.16 | 1.68 | 0.269 | 14/911 | 9/803 | 0.8 | 0.29 | 2.2 | 0.663 |
|  |  | ***≥25*** | 29/887 | 36/832 | 1.28 | 0.73 | 2.24 | 0.389 | 45/887 | 40/832 | 0.92 | 0.55 | 1.57 | 0.771 |
| ***Calcium Channel Blockers*** | ***All women*** |  | 25/1851 | 19/1675 | 1.24 | 0.58 | 2.64 | 0.584 | **27/1851** | **34/1675** | **1.77** | **0.99** | **3.17** | **0.053** |
|  | ***Menopausal **** | ***Premenopausal*** | 2/624 | 1/699 | - | - | - | - | 1/624 | 2/699 | - | - | - | - |
|  |  | ***postmenopausal*** | 22/1213 | 18/976 | 1.61 | 0.72 | 3.61 | 0.248 | 26/1213 | 32/976 | 1.78 | 0.98 | 3.24 | 0.06 |
|  | ***BMI*** | ***<25*** | 6/921 | 4/808 | 1.05 | 0.23 | 4.77 | 0.948 | 13/921 | 6/808 | 0.81 | 0.24 | 2.68 | 0.724 |
|  |  | ***≥25*** | 19/930 | 15/867 | 1.43 | 0.58 | 3.52 | 0.432 | **14/930** | **28/867** | **2.54** | **1.24** | **5.22** | **0.011** |
| ***B- blockers*** | ***All women*** |  | 43/1823 | 31/1660 | 0.88 | 0.52 | 1.48 | 0.622 | 35/1823 | 32/1823 | 1.46 | 0.83 | 2.59 | 0.192 |
|  | ***Menopausal **** | ***Premenopausal*** | 6/619 | 5/695 | 1.11 | 0.28 | 4.35 | 0.882 | 3/619 | 2/695 | - | - | - | - |
|  |  | ***postmenopausal*** | 36/1191 | 26/965 | 0.88 | 0.5 | 1.57 | 0.668 | 31/1191 | 30/965 | 1.54 | 0.84 | 2.8 | 0.159 |
|  | ***BMI*** | ***<25*** | 12/911 | 9/803 | 1.33 | 0.5 | 3.48 | 0.567 | 15/911 | 7/803 | 1.27 | 0.46 | 3.53 | 0.641 |
|  |  | ***≥25*** | 31/912 | 22/857 | 0.7 | 0.37 | 1.32 | 0.27 | 20/912 | 25/857 | 1.58 | 0.78 | 3.23 | 0.208 |
| ***Angiotensin-converting-enzyme inhibitors [ACEIs]*** | ***All women*** |  | 62/1749 | 53/1605 | 0.99 | 0.63 | 1.56 | 0.962 | 74/1749 | 63/1605 | 1.04 | 0.7 | 1.54 | 0.857 |
|  | ***Menopausal **** | ***Premenopausal*** | 6/615 | 9/687 | 1.3 | 0.42 | 3.98 | 0.647 | 2/615 | 5/687 | - | - | - | - |
|  |  | ***postmenopausal*** | 54/1122 | 44/918 | 0.95 | 0.58 | 1.58 | 0.854 | 71/1122 | 58/918 | 1.02 | 0.68 | 1.54 | 0.915 |
|  | ***BMI*** | ***<25*** | 27/887 | 14/795 | 0.88 | 0.38 | 2.05 | 0.77 | 24/887 | 9/795 | 0.47 | 0.19 | 1.15 | 0.1 |
|  |  | ***≥25*** | 35/862 | 39/810 | 1.02 | 0.59 | 1.78 | 0.941 | 50/862 | 54/810 | 1.28 | 0.8 | 2.04 | 0.296 |
| ***Angiotensin II receptor blockers [ARBs]*** | ***All women*** |  | 68/1776 | 59/1607 | 1.15 | 0.75 | 1.76 | 0.533 | 56/1776 | 59/1607 | 1.23 | 0.8 | 1.88 | 0.349 |
|  | ***Menopausal **** | ***Premenopausal*** | 5/620 | 9/683 | 3.48 | 0.86 | 14.02 | 0.079 | 3/620 | 9/683 | - | - | - | - |
|  |  | ***postmenopausal*** | 62/1144 | 50/924 | 1.02 | 0.64 | 1.63 | 0.921 | 53/1144 | 50/924 | 1.07 | 0.68 | 1.67 | 0.771 |
|  | ***BMI*** | ***<25*** | 20/909 | 16/792 | 1.41 | 0.62 | 3.21 | 0.416 | 9/909 | 10/792 | 1.81 | 0.65 | 5.02 | 0.256 |
|  |  | ***≥25*** | 48/867 | 43/815 | 1.05 | 0.63 | 1.75 | 0.848 | 47/867 | 49/815 | 1.1 | 0.69 | 1.76 | 0.685 |

Abbreviations: CI, Confidence interval; OR, odds ratio

^a^OR adjusted for the matching factors age, area of resident, education, body mass index, active smoking, alcohol intake, family history of breast cancer, age of menarche, age first full-term births, number of full-term births, menopausal status, hormonal therapy.

* OR adjusted for the matching factors age, area of resident, education, body mass index, active smoking, alcohol intake, family history of breast cancer, age of menarche, age first full-term births, number of full-term births, hormonal therapy
