# Supplementary material for: The Use of Antihypertensive Medication and the Risk of Breast Cancer in a Case-Control Study in a Spanish Population: The MCC-Spain Study
Source: PLoS One. 2016 Aug 10;11(8):e0159672. doi: 10.1371/journal.pone.0159672 (PMC4979888; doi:10.1371/journal.pone.0159672)
Supplement: S2 Table — Category reference no antihypertensive treatment. (DOCX) [file pone.0159672.s002.docx]

**S2 Table. Association between duration of antihypertensive drug consumption (<5 years and ≥5 years) and the risk of breast cancer according to characteristics of tumor and immunohistochemistry. Category reference no antihypertensive treatment**

|  |  |  | **Population Controls** | **Breast Cancer Cases if antihypertensive therapy use <5 years** | | | | | **Population Controls** | **Breast Cancer Cases if antihypertensive therapy use ≥5 years** | | | | |
| --- | --- | --- | --- | --- | --- | --- | --- | --- | --- | --- | --- | --- | --- | --- |
|  |  |  | **Exp /UnExp** | **Exp /UnExp** | ***Adjusted OR*** | ***95% CI*** | | ***p-value*** | **Exp /UnExp** | **Exp /UnExp** | ***Adjusted OR*** | ***95% CI*** | | ***p-value*** |
| ***any antihypertensive therapy*** | ***Clinical Stage*** | **I-II** | 142/1497 | 86/872 | 1.35 | 0.96 | 1.89 | 0.082 | 225/1497 | 122/872 | 1.14 | 0.85 | 1.52 | 0.385 |
|  |  | **III-IV** | 142/1497 | 21/154 | 1.79 | 0.98 | 3.25 | 0.056 | 225/1497 | 26/154 | 1.48 | 0.86 | 2.53 | 0.153 |
|  | ***ductal*** | **Ductal** | 142/1497 | 105/1015 | 1.35 | 0.97 | 1.87 | 0.072 | 225/1497 | 140/1015 | 1.13 | 0.85 | 1.5 | 0.395 |
|  |  | **Non ductal** | 142/1497 | 20/177 | 1.58 | 0.91 | 2.75 | 0.108 | 225/1497 | 30/177 | 1.42 | 0.87 | 2.31 | 0.165 |
|  | ***Invasive*** | **In situ** | 142/1497 | 6/143 | 0.62 | 0.26 | 1.49 | 0.282 | 225/1497 | 15/143 | 0.63 | 0.31 | 1.3 | 0.212 |
|  |  | **Invasive** | **142/1497** | **121/1172** | **1.38** | **1.01** | **1.89** | **0.04** | 225/1497 | 168/1172 | 1.18 | 0.9 | 1.54 | 0.229 |
|  | ***Inmunohistochemistry*** | **hormone +receptors** | 142/1497 | 84/884 | 1.15 | 0.81 | 1.62 | 0.433 | 225/1497 | 126/884 | 1.12 | 0.83 | 1.49 | 0.461 |
|  |  | **Erbb2 +receptors** | 142/1497 | 17/205 | 1.28 | 0.72 | 2.27 | 0.404 | 225/1497 | 28/205 | 1.04 | 0.61 | 1.76 | 0.894 |
|  |  | **triple negative receptors** | **142/1497** | **21/111** | **2.74** | **1.48** | **5.06** | **0.001** | **225/1497** | **20/111** | **1.91** | **1.07** | **3.49** | **0.028** |
| ***Diuretics*** | ***Clinical Stage*** | **I-II** | 45/1798 | 26/1039 | 1.09 | 0.63 | 1.92 | 0.753 | 59/1798 | 29/1039 | 0.75 | 0.43 | 1.31 | 0.309 |
|  |  | **III-IV** | 45/1798 | 6/193 | 1.32 | 0.5 | 3.51 | 0.58 | 59/1798 | 5/193 | 0.76 | 0.26 | 2.22 | 0.621 |
|  | ***ductal*** | **Ductal** | 45/1798 | 34/1209 | 1.16 | 0.69 | 1.97 | 0.575 | 59/1798 | 38/1209 | 0.93 | 0.56 | 1.53 | 0.764 |
|  |  | **Non ductal** | 45/1798 | 5/223 | 1.01 | 0.38 | 2.68 | 0.988 | 59/1798 | 5/223 | 0.59 | 0.2 | 1.72 | 0.336 |
|  | ***Invasive*** | **In situ** | 45/1798 | 2/161 | - | - | - | - | 59/1798 | 3/161 | - | - | - | - |
|  |  | **Invasive** | 45/1798 | 40/1406 | 1.2 | 0.73 | 1.98 | 0.474 | 59/1798 | 42/1406 | 0.87 | 0.53 | 1.41 | 0.57 |
|  | ***Inmunohistochemistry*** | **hormone +receptors** | 45/1798 | 26/1054 | 0.98 | 0.56 | 1.73 | 0.952 | 59/1798 | 30/1054 | 0.84 | 0.49 | 1.44 | 0.531 |
|  |  | **Erbb2 +receptors** | 45/1798 | 7/240 | 1.49 | 0.64 | 3.5 | 0.357 | 59/1798 | 7/240 | 0.67 | 0.23 | 1.94 | 0.463 |
|  |  | **triple negative receptors** | 45/1798 | 8/144 | 2.31 | 0.96 | 5.52 | 0.06 | 59/1798 | 5/144 | 0.98 | 0.33 | 2.85 | 0.964 |
| ***Calcium Channel Blockers*** | ***Clinical Stage*** | **I-II** | 25/1851 | 9/1065 | 0.93 | 0.37 | 2.36 | 0.878 | 27/1851 | 20/1065 | 1.63 | 0.85 | 3.13 | 0.139 |
|  |  | **III-IV** | 25/1851 | 5/193 | 2.92 | 0.91 | 9.37 | 0.072 | 27/1851 | 6/193 | 2.59 | 0.93 | 7.2 | 0.069 |
|  | ***ductal*** | **Ductal** | 25/1851 | 16/1248 | 1.4 | 0.63 | 3.13 | 0.41 | 27/1851 | 22/1248 | 1.57 | 0.82 | 3.01 | 0.172 |
|  |  | **Non ductal** | 25/1851 | 2/218 | - | - | - | - | **27/1851** | **10/218** | **3.96** | **1.73** | **9.05** | **0.001** |
|  | ***Invasive*** | **In situ** | 25/1851 | 1/163 | - | - | - | - | 27/1851 | 2/163 | - | - | - | - |
|  |  | **Invasive** | 25/1851 | 17/1441 | 1.2 | 0.58 | 2.8 | 0.543 | **27/1851** | **32/1441** | **1.96** | **1.09** | **3.53** | **0.025** |
|  | ***Inmunohistochemistry*** | **hormone +receptors** | 25/1851 | 11/1080 | 0.97 | 0.4 | 2.39 | 0.952 | 27/1851 | 22/1080 | 1.82 | 0.96 | 3.46 | 0.068 |
|  |  | **Erbb2 +receptors** | 25/1851 | 4/242 | - | - | - | - | **27/1851** | **8/242** | **2.97** | **1.2** | **7.32** | **0.019** |
|  |  | **triple negative receptors** | 25/1851 | 2/152 | - | - | - | - | 27/1851 | 2/152 | - | - | - | - |
| ***B- blockers*** | ***Clinical Stage*** | **I-II** | 43/1823 | 21/1054 | 0.99 | 0.56 | 1.76 | 0.969 | 35/1823 | 19/1054 | 1.27 | 0.66 | 2.43 | 0.471 |
|  |  | **III-IV** | 43/1823 | 5/194 | 0.84 | 0.25 | 2.83 | 0.777 | 35/1823 | 4/194 | 1 | 1 | 1 | . |
|  | ***ductal*** | **Ductal** | 43/1823 | 27/1229 | 1.06 | 0.61 | 1.84 | 0.832 | 35/1823 | 21/1229 | 1.29 | 0.68 | 2.45 | 0.44 |
|  |  | **Non ductal** | 43/1823 | 2/225 | - | - | - | - | 35/1823 | 6/225 | 1.96 | 0.77 | 5.01 | 0.158 |
|  | ***Invasive*** | **In situ** | 43/1823 | 1/162 | - | - | - | - | 35/1823 | 3/162 | - | - | - | - |
|  |  | **Invasive** | 43/1823 | 28/1429 | 0.98 | 0.57 | 1.68 | 0.943 | 35/1823 | 27/1429 | 1.43 | 0.79 | 2.6 | 0.234 |
|  | ***Inmunohistochemistry*** | **hormone +receptors** | 43/1823 | 21/1073 | 0.87 | 0.48 | 1.57 | 0.635 | 35/1823 | 17/1073 | 1.1 | 0.55 | 2.19 | 0.791 |
|  |  | **Erbb2 +receptors** | 43/1823 | 3/246 | - | - | - | - | 35/1823 | 3/246 | - | - | - | - |
|  |  | **triple negative receptors** | 43/1823 | 4/146 | - | - | - | - | **35/1823** | **5/146** | **3.03** | **1.08** | **8.45** | **0.035** |
| ***Angiotensin-converting-enzyme inhibitors [ACEIs]*** | ***Clinical Stage*** | **I-II** | 62/1749 | 35/1015 | 1.1 | 0.67 | 1.81 | 0.712 | 74/1749 | 42/1015 | 1.07 | 0.68 | 1.67 | 0.772 |
|  |  | **III-IV** | 62/1749 | 8/189 | 1.1 | 0.42 | 2.91 | 0.842 | 74/1749 | 6/189 | 0.96 | 0.4 | 2.32 | 0.925 |
|  | ***ductal*** | **Ductal** | 62/1749 | 40/1196 | 0.95 | 0.57 | 1.56 | 0.828 | 74/1749 | 43/1196 | 0.95 | 0.61 | 1.48 | 0.816 |
|  |  | **Non ductal** | 62/1749 | 11/209 | 1.97 | 0.96 | 4.03 | 0.064 | 74/1749 | 11/209 | 1.58 | 0.79 | 3.16 | 0.196 |
|  | ***Invasive*** | **In situ** | 62/1749 | 2/157 | - | - | - | - | 74/1749 | 7/157 | 1.18 | 0.45 | 3.12 | 0.734 |
|  |  | **Invasive** | 62/1749 | 49/1380 | 1.25 | 0.81 | 1.95 | 0.314 | 74/1749 | 55/1380 | 1.1 | 0.73 | 1.66 | 0.646 |
|  | ***Inmunohistochemistry*** | **hormone +receptors** | 62/1749 | 39/1025 | 1.19 | 0.73 | 1.96 | 0.488 | 74/1749 | 46/1025 | 1.17 | 0.75 | 1.82 | 0.488 |
|  |  | **Erbb2 +receptors** | 62/1749 | 4/242 | - | - | - | - | 74/1749 | 8/242 | 0.95 | 0.42 | 2.17 | 0.901 |
|  |  | **triple negative receptors** | 62/1749 | 7/139 | 1.36 | 0.5 | 3.68 | 0.541 | 74/1749 | 7/139 | 1.51 | 0.64 | 3.54 | 0.343 |
| ***Angiotensin II receptor blockers [ARBs]*** | ***Clinical Stage*** | **I-II** | 68/1776 | 36/1019 | 1.06 | 0.64 | 1.74 | 0.839 | 56/1776 | 40/1019 | **1.66** | **1.05** | **2.63** | **0.031** |
|  |  | **III-IV** | 68/1776 | 11/184 | **2.17** | **1.03** | **4.53** | **0.04** | 56/1776 | 8/184 | 1.31 | 0.53 | 3.24 | 0.558 |
|  | ***ductal*** | **Ductal** | 68/1776 | 50/1189 | 1.34 | 0.85 | 2.11 | 0.206 | 56/1776 | 41/1189 | 1.22 | 0.77 | 1.95 | 0.402 |
|  |  | **Non ductal** | 68/1776 | 7/218 | 0.98 | 0.41 | 2.39 | 0.971 | 56/1776 | 9/218 | 1.48 | 0.69 | 3.18 | 0.313 |
|  | ***Invasive*** | **In situ** | 68/1776 | 1/155 | - | - | - | - | 56/1776 | 8/155 | 1.18 | 0.44 | 3.18 | 0.738 |
|  |  | **Invasive** | 68/1776 | 55/1384 | 1.25 | 0.81 | 1.95 | 0.314 | 56/1776 | 49/1384 | 1.23 | 0.79 | 1.91 | 0.369 |
|  | ***Inmunohistochemistry*** | **hormone +receptors** | 68/1776 | 35/1039 | 0.94 | 0.57 | 1.56 | 0.807 | 56/1776 | 39/1039 | 1.28 | 0.8 | 2.05 | 0.296 |
|  |  | **Erbb2 +receptors** | 68/1776 | 8/234 | 1.28 | 0.55 | 2.96 | 0.564 | 56/1776 | 11/234 | 1.63 | 0.76 | 3.52 | 0.211 |
|  |  | **triple negative receptors** | 68/1776 | 11/142 | **2.48** | **1.14** | **5.41** | **0.023** | 56/1776 | 3/142 | - | - | - | - |

Abbreviations: CI, Confidence interval; OR, odds ratio

^a^OR adjusted for the matching factors age, area of resident, education, body mass index, active smoking, alcohol intake, family history of breast cancer, age of menarche, age first full-term births, number of full-term births, menopausal status, hormonal therapy.

* OR adjusted for the matching factors age, area of resident, education, body mass index, active smoking, alcohol intake, family history of breast cancer, age of menarche, age first full-term births, number of full-term births, hormonal therapy
